# Lagrangian Differencing Dynamics for Time-Independent Non-Newtonian Materials

**DOI:** 10.3390/ma14206210

**Published:** 2021-10-19

**Authors:** Martina Bašić, Branko Blagojević, Chong Peng, Josip Bašić

**Affiliations:** 1 Faculty of Electrical Engineering, Mechanical Engineering and Naval Architecture, University of Split, R. Boškovića 32, 21000 Split, Croatia; bblag@fesb.hr (B.B.); jobasic@fesb.hr (J.B.); 2 Institute of Geotechnical Engineering, University of Natural Resources and Life Sciences Vienna, Feistmantelstraße 4, 1180 Wien, Austria; pengchong@boku.ac.at

**Keywords:** non-Newtonian, rheology, Lagrangian, meshless, LDD, Casson, Bingham, Power Law

## Abstract

This paper introduces a novel meshless and Lagrangian approach for simulating non-Newtonian flows, named Lagrangian Differencing Dynamics (LDD). Second-order-consistent spatial operators are used to directly discretize and solve generalized Navier–Stokes equations in a strong formulation. The solution is obtained using a split-step scheme, i.e., by decoupling the solutions of the pressure and velocity. The pressure is obtained by solving a Poisson equation, and the velocity is solved in a semi-implicit formulation. The matrix-free solution to the equations, and Lagrangian advection of mesh-free nodes allowed for a fully parallelized implementation on the CPU and GPU, which ensured an affordable computing time and large time steps. A set of four benchmarks are presented to demonstrate the robustness and accuracy of the proposed formulation. The tested two- and three-dimensional simulations used Power Law, Casson and Bingham models. An Abram slump test and a dam break test were performed using the Bingham model, yielding visual and numerical results in accordance with the experimental data. A square lid-driven cavity was tested using the Casson model, while the Power Law model was used for a skewed lid-driven cavity test. The simulation results of the lid-driven cavity tests are in good agreement with velocity profiles and stream lines of published reports. A fully implicit scheme will be introduced in future work. As the method precisely reproduces the pressure field, non-Newtonian models that strongly depend on the pressure will be validated.

## 1. Introduction

Various types of fluids used in industry and in nature do not act in accordance with Newton’s law, assuming that the fluid viscosity is constant. Non-Newtonian flows include mud, paint, blood, lymph fluid, cell fluid, milk, chocolate, edible oil, etc. Non-Newtonian fluids exhibit viscous properties on a regular basis, and it is important for the designer or engineer to be familiar with the flow behavior of such fluids, be able to identify the fluids’ physical properties, and know how to use these properties to predict flow behavior in the industrial process. Viscoplastic and viscous fluids are both time-independent fluid categories, and novel numerical models belonging to these classes of non-Newtonian fluids will be discussed in this paper.

Purely viscous models simulate shear-thickening and shear-thinning fluids. Shear-thinning fluids can be described by the Casson model [1], while the Power Law model [2] is a more generalized model. It is assumed that the Casson model has infinite viscosity for a zero shear rate, a yield stress below which the fluid does not flow, and zero value of the viscosity at an infinite shear rate. Shear-thinning behavior describes the melting of polymers, polymer solutions, biological fluids, mud and mayonnaise. The Power Law is a widely used, mathematically simple model that can approximately simulate the behavior of a non-Newtonian fluid. Depending on the flow-behavior index *n*, it can be classified into three types of fluids. For n<1, the effective viscosity decreases with an increase in shear rate, i.e., it describes the shear-thinning fluid. For n>1, the model describes a shear-thickening fluid, and n=1 describes a Newtonian fluid. The Bingham model [3,4] is also a widely used, viscoplastic, non-Newtonian model. This viscoplastic model is commonly used in engineering due to its mathematical simplicity, i.e., it is a two-parameter model. It is used in food, drilling, oil and gas, chemical and many other industries, as the majority of industrial fluids comply with Bingham’s law. Depending on the stress level, Bingham fluid acts either as a rigid body or a viscous fluid. The yield stress must be exceeded for the fluid to flow. When the initial stress is applied, the fluid will not start to flow immediately. The shear stress must be raised until the yield stress of the fluid is reached. This is contrary to Newtonian fluids, which have zero yield stress and can flow instantly. The Bingham model is used to model debris flows and landslides, lubrication, mud flows, fresh cement, snow avalanches, lava flows, etc.

Non-Newtonian flow modeling is used in a wide range of applications, including industrial, mechanical, and medical applications. The Finite Element Method (FEM) was mostly used to investigate viscoplastic flows, while viscoelastic fluids are challenging to simulate due to the loss of convergence. Fortin and Fortin [5] investigated numercial schemes for high Deborah numbers (De). The De number is a dimensionless number used to describe the fluidity of the flow as a ratio of the time needed for a fluid to adjust to the applied stress and time scale of the experiment, or a computer simulation. Szady et al. [6] introduced a new discrete elastic-viscous-split-stress FEM method, which increases numerical stability for De > 100. Grillet et al. [7], in their study, used mixed (DEVSS/hp-SUPG) FEM to simulate the effect of fluid elasticity and stress distribution in lid-driven cavity flow. Křen and Hynčík [8] derived basic continuity and Navier–Stokes equations for Newtonian fluid, using of special constitutive equations for viscosity to solve the non-Newtonian fluid flow. Convection in a square cavity with the Bingham model, without regularization, was tested in the work of Huilgol and Kefayati [9]. Celigueta et al. [10] presented a procedure to couple the FEM for Eulerian and particle FEM (PFEM) for Lagrangian flows with the discrete-element method (DEM) for the cutting transport process.

In his paper, Neofytou [11] researched non-Newtonian flow effects using generalized Newtonian constitutive equations with the Finite-Volume Method (FVM). The lid-driven cavity flow was performed for Newtonian and various non-Newtonian flows. Zou et al. [12] incorporated the Latice Boltzmann method (LBM) with the FVM and proposed an integration scheme for incompressible viscoelastic fluid flow. De et al. [13] simulated unsteady non-Newtonian flow on the 3D porous medium, employing FVM with staggered grid. De et al. [14] researched the creeping flow of a viscoelastic fluid through a porous 3D medium using FVM-IBM.

Unlike the abovementioned Eulerian methods, in Lagrangian methods, the convective term is not included in the momentum equation, so there is no need for numerical stabilization. When large deformations are expected, the Lagrangian approach preferable to Eulerian mesh methods. Salazar et al. [15] used the PFEM to model the fluid–structure interaction for landslides. Landslides generate impulse waves and, due to the uncertain kinematics of the mobilized material, it is difficult to calculate fluid–solid interactions. Cremonesi et al. [16] conducted a number of tests on Newtonian and non-Newtonian fluids in order to validate the PFEM method. Larese [17] introduced a stabilized–mixed PFEM for the calculation of non-Newtonian viscoplastic flows. Franci and Zhang [18] simulated free-surface Bingham fluids using the Lagrangian approach. Two- and three-dimensional simulations were carried out using the PFEM, and solid structures were simulated by employing FEM. Della Vecchia et al. [19] investigated Bingham fluids, specifically the rheological characteristics of water–soil mixtures on the dam-break tests. The numerical analysis examined the viscosity and yield stress of the Bingham model based on the CFD-PFEM parametric studies. Marchi et al. [20] obtained numerical PFEM solutions for a laminar flow within a square cavity, with a variable velocity lid, for which the analytical solution is known, and for laminar flow inside a square cavity with a constant velocity lid, for which the analytical solution is unknown.

In Bognár and Csáti [21] paper, a spectral approach is used to address the lid-driven cavity flow problem and develop a MATLAB software to solve the Navier–Stokes equation. Bruneau and Saad [22] researched Hopf bifurcation, and the solution’s behavior for intermediate and high Reynolds numbers, based on a finite-differences (FD) discretization and a multigrid solver with cell-by-cell relaxation. The findings of Coclite et al. [23] and Coclite [24], using time LBM in the rheology of elliptical particles, can provide specific insight into the rational design of micro- and nano-particles as drug carriers.

The Smoothed Particle Hydrodynamics (SPH) method is a Lagrangian and meshless method, which is widely used for a variety of fluid flow simulations [25,26,27]. Researchers and scientists are continually developing the SPH method, solvers and models according to the type of flow, although its traditional formulation suffers from an inaccurate pressure field [28]. The SPH has also been applied to non-Newtonian free-surface problems, such as mud and molding flows [29,30]. Hosseini et al. [31] presented a GPU implementation to achieve a better performance. Shao and Lo [30] simulated a dam-break problem and discussed the flow features of Newtonian and non-Newtonian flows using the Incompressible SPH (ISPH). Its advantage lies in the ease of tracking the free surface using a similar procedure to that employed in the the moving particle semi-implicit (MPS) method, and the findings correlated well with the experiments. Fan et al. [32] developed a matrix-free, implicit SPH solver for highly viscous non-Newtonian flows that contain high-pressure areas. The standard, explicit SPH method was not feasible due to the need for a very small time step to achieve a stable simulation. Zhu et al. [33] assessed how well the plastic viscosity can be determined using the SPH approach. Papanastasiou’s Bingham constitutive model [34] was implemented in the SPH model and tested against the results of published data. Xu et al. [35] enhanced the SPH approach to 3D non-Newtonian flows with complex free-surface shapes and the Casson model. An artificial stress-term is inserted into the momentum equation to prevent tensile instability, which leads to the clustering of particles and non-physical defects in fluid stretching. Xenakis et al. [28] used a diffusion-based ISPH method to describe free-surface flows. The approach was developed to solve inelastic non-Newtonian flows by introducing a new viscous term that is more suitable for such flows.

The objective of the present work is to introduce a Lagrangian Differencing Dynamics (LDD) method for the simulation of non-Newtonian fluids, which combines the advantages of the meshless Lagrangian methods, such as SPH and MPS, with the advantages of implicit schemes, such as FVM or FEM. Peng et al. [36] applied LDD to granular flow modeling using a Drucker–Prager model, and found that its accuracy in terms of pressure and impact force is higher than SPH. This success promoted the idea of applying the LDD to simulate a wide range of non-Newtonian materials in this work. The LDD method relies on second-order consistent operators introduced by Basic et al. [37], and extends the scheme introduced by Bašić et al. [38]. The scheme solves the Navier–Stokes equations (NSE) using the split-step approach, where the pressure field is solved implicitly, and the velocity is solved explicitly. On the other hand, in this work, the scheme is extended to solve generalized NSE with variable viscosity, while solving the momentum equation in a semi-implicit manner. After obtaining the pressure and velocity fields, the LDD method relies on Lagrangian advection, which is shown to enable large time steps.

In this paper, the authors reproduced four benchmarks from different authors to validate the proposed methodology. The original benchmarks reproduced in this paper used different numerical methods and mathematical approaches to solve a specific set of problems for non-Newtonian and Newtonian fluids. The validation tests replicated in this study using the LDD method are as follows: dam break case originally validated using SPH and Bingham model [28], square lid-driven cavity simulated using the FVM and Casson model [11], skewed lid-driven cavity flow simulated using the FVM method and Power Law model [39] and fresh concrete Abram slump test problem validated using the PFEM and Bingham model [18].

The remainder of the paper is organized as follows. In Section 2, the mathematical model for simulating various viscoplastic materials is presented. Section 3.1 explains the numerical procedure for solving the introduced mathematical system. Section 4 presents the validation cases and discusses the numerically obtained results. Finally, the conclusions are drawn in Section 5.

## 2. Governing Equations

The generalized Navier–Stokes equations (NSE), in vector form, for the non-Newtonian incompressible fluid flow are described and solved. The conservation of momentum and mass is given as follows:

(1) $$\frac{D(\rho u)}{Dt}=-\nabla p+\nabla \cdot \tau +F_{ext},$$

(2) ∇·u=0,

where the advective derivative is expressed as D/Dt, the velocity vector as **u**, the fluid density as ρ, the fluid pressure as *p*, the stress tensor as τ, and $F_{ext}$ as the vector of external forces. Equations (1) and (2) imply temporal and spatial dependency. The stress tensor τ is defined for an incompressible fluid using the following expression:

(3) $$\tau =2\;\mu \left(E\right)\;E,$$

where μ is the dynamic viscosity of the fluid, and E is the strain rate tensor, which is defined as:

(4) $$E=\frac{1}{2}\left(\nabla u+{\left(\nabla u\right)}^{T}\right),$$

where **∇u** indicates the velocity-gradient tensor of the flowing material. The shear rate is defined as:

(5) $$\dot{\gamma }=\sqrt{2E:E^{T}},$$

where the colon (or double-dot) operator is defined as $E:E^{T}\equiv trace\left(EE^{T}\right)$, and trace represents the sum of the matrix diagonal elements.

### 2.1. Bingham Model

The Bingham model is a widely used, viscoplastic non-Newtonian model. This viscoplastic model is commonly used in engineering due to its mathematical simplicity, i.e., it is a two-parameter model. It is used in food, drilling, oil and gas, chemical and many other industries, as the majority of industrial fluids comply with Bingham’s law. According to the Papanastasiou [34] model, the stress tensor is calculated as:

(6) $$\tau =\left[\mu_{\infty }+\frac{\tau_{0}}{\left|\dot{\gamma }\right|}\left(1-e^{-m\left|\dot{\gamma }\right|}\right)\right]\dot{\gamma },$$

where $\tau_{0}$is the yield stress, $\mu_{\infty }$ is the dynamic viscosity at infinite shear rate, $\dot{\gamma }=2E$, and *m* is the regularization parameter. The effective viscosity is calculated as follows:

(7) $$\mu \left(\left|\dot{\gamma }\right|\right)=\mu_{\infty }+\frac{\tau_{0}}{\left|\dot{\gamma }\right|}\left(1-e^{-m\left|\dot{\gamma }\right|}\right).$$

### 2.2. Casson Model

The Casson model is a rheological model that is used to describe viscoelastic flow. It is expressed in accordance with the Papanastasiou [34] regularization:

(8) $$\tau ={\left[{\sqrt{\mu }}_{\infty }+\sqrt{\frac{\tau_{0}}{\left|\dot{\gamma }\right|}}\left(1-e^{-\sqrt{m\left|\dot{\gamma }\right|}}\right)\right]}^{2}\dot{\gamma },$$

where $\tau_{0}$ is the yield stress, $\mu_{\infty }$ is the dynamic viscosity at infinite shear rate and *m* is the regularization parameter. The effective viscosity is obtained as follows:

(9) $$\mu \left(\left|\dot{\gamma }\right|\right)={\left[{\sqrt{\mu }}_{\infty }+\sqrt{\frac{\tau_{0}}{\left|\dot{\gamma }\right|}}\left(1-e^{-\sqrt{m\left|\dot{\gamma }\right|}}\right)\right]}^{2}$$

### 2.3. Power Law Model

Power Law is a widely used and mathematically simple model that can approximately simulate the behavior of a non-Newtonian fluid. In this generalized model, for purely viscous fluids, shear stress tensor is calculated as:

(10) $$\tau =\mu_{0}{\left|\dot{\gamma }\right|}^{n-1}\dot{\gamma },$$

the generalized model of the Power Law is defined by the effective viscosity in function of the shear rate, as follows:

(11) $$\mu \left(\left|\dot{\gamma }\right|\right)=\mu_{0}{\left|\dot{\gamma }\right|}^{n-1},$$

where $\mu_{0}$ represents the flow consistency index, and *n* is the flow behavior index. Depending on the flow-behavior index n, it can mathematically model three types of fluids. The fluid describes shear-thinning flows for 0<n<1, Newtonian behavior for n=1, and shear-thickening flows for n>1. The zero-shear viscosity is approached at very low shear rates, while the infinite shear viscosity is approached at very high shear rates.

## 3. Numerical Procedure

### 3.1. Lagrangian Differencing

**Figure 1:**
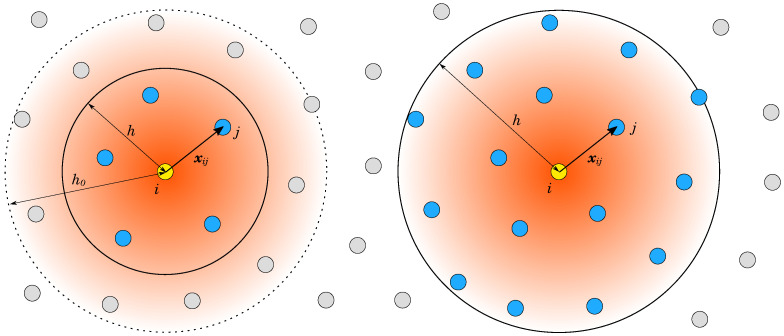
Neighbor interaction in LDD (**left image**) versus SPH and MPS (**right image**). The set of neighboring nodes N found in the compact sphere (blue) with radius *h* and centered at location $x_{i}$. The gradient depicts weighting of the interaction based on the distance from $x_{i}$.

The defined fluid domain is represented without topological information, using a cloud of points (i.e., nodes) described by the set Ω. At a given time, each node i∈Ω at location $x_{i}\left(t\right)$ in a specific time instant *t* carries some fluid properties. Each node *i* interacts with a set of neighbor nodes, N, which are found in the sphere with radius *h*, as shown in Figure 1. A symmetric and positive weighting function $W\left(r,\;h_{0}\right)$, is used to evaluate the strength of the interaction between nodes, based on the distance between them, where $r=∥x_{i}-x_{j}∥$. The weighting function radius is represented by $h_{0}$, and when $r\geq h_{0}$, the weighting function equals zero. The weighting function is defined as $W\left(r,\;h_{0}\right)=(1-r/h_{0}$)${}^{3}$, where $0\leq r\leq h_{0}$. Unlike in the SPH method, where the particles are constrained to a specific volume and the smoothing function must fulfill the quadrature requirements [27], in this study the weighting function is used only for the weighting of interactions in the finite-difference manner; therefore, it can take an arbitrary shape. This so-called Lagrangian differencing only accounts for a small number of immediate neighboring nodes [36], because a high accuracy is guaranteed due to the renormalization, which is introduced below. Consequently, the introduced method is more efficient when compared to classical SPH and MPS methods [36,38], which often employ a large compact radius to encapsulate a large number of neighbors because the convergence requirements of SPH approximation include particle spacing Δ→0, and ratio h/Δ→0 [36]. In the LDD method, only the nearest neighbors need to be accounted for; therefore, h/Δ is taken as 1.6, significantly below the SPH standards.

Values of some continuous function $f\left(x\right)$ may be approximated anywhere between nodes by using Shepard’s formula:

(12) $$\langle f\left(x\right)\rangle =\frac{\sum_{j\in N}W_{j}\;f_{j}}{\sum_{j\in N}W_{j}},$$

where 〈〉 indicates a discrete version of some expression, $f_{j}\equiv f\left(x_{j}\right)$ is introduced for compactness, and $W_{j}\equiv W\left(∥x-x_{j}∥,\;h_{0}\right)$ indicates the weight of the neighbor node *j* in the neighborhood. A renormalization tensor $B_{i}$ for the discrete gradient is defined as [37]:

(13) $$B_{i}={\left(\sum_{j\in N}W_{ij}\;x_{ij}\;x_{ij}^{T}\right)}^{-1},$$

where $W_{ij}\equiv W\left(∥x_{j}-x_{i}∥,\;h_{0}\right)$ and $x_{ij}\equiv x_{j}-x_{i}$. Using Equation (13), the discrete approximation of the gradient is obtained using the expression:

(14) $${\langle \nabla f\rangle }_{i}=B_{i}\sum_{j\in N}W_{ij}\;f_{ij}\;x_{ij}.$$

The derivation of this expression for the gradient is given in [37], and the convergence theorems are presented in [40]. The method was validated to be consistent up to the second order, and robust for highly irregular arrangements of nodes.

An accurate discrete expression for the Laplacian operator used in this study belongs to a class of renormalized Laplacian operators introduced by Basic et al. [37], which were validated on particularly irregular neighborhood arrangements. The discrete Laplacian is defined as:

(15) $${\langle \nabla^{2}f\rangle }_{i}=2d\frac{\sum_{j\in N}W_{ij}\;f_{ij}\left(1-x_{ij}\cdot B_{i}o_{i}\right)}{\sum_{j\in N}W_{ij}\;{∥x_{ij}∥}^{2}\left(1-x_{ij}\cdot B_{i}o_{i}\right)},$$

where $o_{i}$ is the offset vector of the node *i*:

(16) $$o_{i}=\sum_{j\in N}W_{ij}\;x_{ij},$$

which points from $x_{i}$ to the point where the arrangement of the neighborhood dominates. Bašić et al. [38] have shown that the renormalization process enhances the operator when approximating a scalar field Laplacian, and that it is responsible for reaching second-order accuracy when solving Poisson problems, while the original SPH and MPS formulations yield first-order accuracy. This operator is a crucial ingredient in discretizing the pressure and velocity equations.

### 3.2. Numerical Solver

In the split-step scheme, the pressure and velocity steps are solved separately. The pressure is obtained by solving the Poisson equation within the proper boundary conditions, and the solution must satisfy the continuity constraint. The pressure Poisson equation (PPE) is defined as:

(17) $$\begin{array}{c} \left\{\begin{array}{cc} \nabla^{2}p=\rho \;\nabla \cdot \left[\nabla \cdot \tau -Du/Dt\right] & x\in \Omega ,\\ n\cdot \nabla p=\rho \;n\cdot \left[-\partial_{t}U+F_{ext}-\nu \nabla \times \nabla \times u\right] & x\in \Gamma_{w},\\ p=p_{atm} & x\in \Gamma_{fs}, \end{array}\right. \end{array}$$

where n is the normal boundary. In Equation (17) the no-slip boundary condition along the $\Gamma_{w}$ interface is obtained by dotting Equation (1) with n. The external acceleration is taken as a constant; therefore, $\nabla \cdot F_{ext}=0$ is excluded from Equation (17). Furthermore, ∇·∇·τ is also taken as zero in Equation (17), since it was empirically tested and did not significantly affect the simulated problems. In other words, the spatial change in the stress divergence vector field does not generate a significant pressure gradient or affect the flowing material. This does not hold in general, but holds for the types of flows simulated in this study. The general impact of the term ∇·∇·τ on the pressure and velocity will be analyzed in future work. The PPE was solved using the BiCGStab iterative solver in the matrix-free manner, as described by Bašić et al. [38]. After solving the PPE (17), the pressure gradient may be explicitly calculated using Equation (14), and the results may be substituted into the momentum Equation (1). The shear rate was calculated using Equation (5), also based on explicitly calculated velocity gradients for the strain rate Equation (4) using Equation (14).

At this point, the velocity vector is the only unknown in the momentum Equation (1). Since the stress tensor τ is defined by Equation (3) through the strain rate E (Equation (4)), the stress tensor divergence may be written as $\nabla \cdot \tau =\mu \;\nabla^{2}u+2E\;\nabla \mu$, and substituted back into Equation (1), which can be stated as:

(18) $$\rho \frac{Du}{Dt}=-\nabla p+\mu \nabla^{2}u+2E\;\nabla \mu +F_{ext},$$

In this study, the momentum equation is solved for velocity in the semi-implicit formulation. The term with the velocity Laplacian is placed on the left-hand-side of the equation, while the term with the viscosity gradient is explicitly evaluated and placed on the right-hand-side. Furthermore, the Lagrangian acceleration term is discretized by the Euler approximation. The problem is, therefore, defined by the following expression:

(19) $$\rho u_{n}-\mu \nabla^{2}u_{n}=\rho u_{n-1}+\delta t\left\{-\nabla p+F_{ext}+2E_{n-1}\nabla \mu \right\},$$

where *n* denotes the step with unknown variables, and n−1 denotes the previous time-step with known values. The numerical method is built by directly discretizing equations using the discrete spatial operators described in Section 3.1. The analyses given in Basic et al. [37] have shown that the types of problems described using Equations (17) and (19) reach second-order convergence for Dirichlet, Neumann and mixed-boundary conditions. Equation (19), along with the no-slip boundary condition, can be straightforwardly solved using an iterative matrix-free solver.

After obtaining the velocity field by solving $u_{n}$ in Equation (19), mesh-free nodes are advected in the Lagrangian manner. Second-order Euler approximation is applied to advance positions of mesh-free nodes, using the velocities of the previous and currently obtained time-steps. Therefore, the newly advected position of a node *i* is explicitly obtained by calculating the expression:

(20) $$x_{i,n+1}=x_{i,n}+\left[\left(3/2\right)u_{i,n}-\left(1/2\right)u_{i,n-1}\right]\delta t.$$

The Lagrangian solution dictates that each node advects along its streamline. Since the size of the time-step is finite and the flow is often complex, advecting along the streamline tangent can lead to the eventual separation or collision of nodes’ trajectories. The possibility of distorted node arrangements and errors in conservation of the volume are resolved by introducing a reordering step after the advection of mesh-free node neighbourhoods.

The reordering step employs the Position-Based Dynamics (PBD) method introduced by Macklin and Müller [41], which iteratively displaces node neighborhoods to enforce constant density in the fluid. This results in equally spaced nodes in the point cloud, thereby enforcing incompressibility and allowing the utilization of larger time-steps. The velocities at reordered locations are interpolated using Equation (12) only for excessive time-step sizes and significant reorderings, as described by Bašić et al. [38]. Generally, using reasonable time-step sizes does not result in significant reordering, but a minor movement of the node position within its radius to maintain the equidistant property.

**Figure 2:**
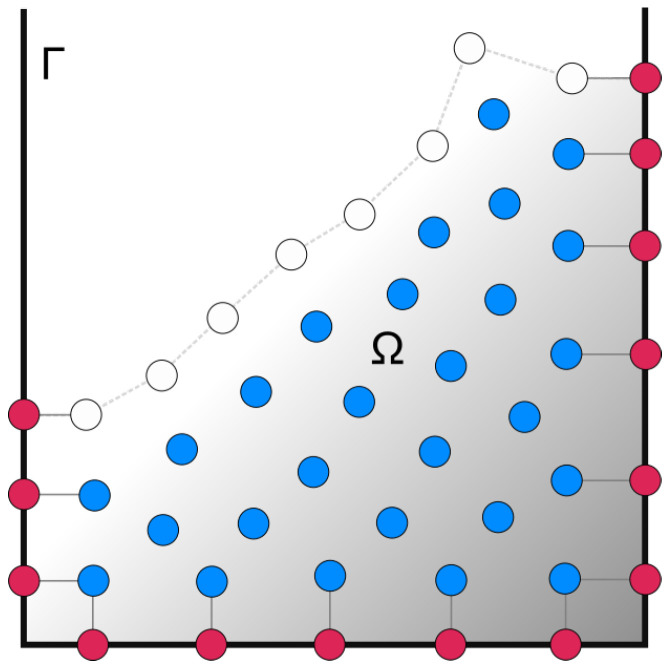
Inner fluid nodes (blue) and boundary conditions, i.e., wall nodes (red) and free surface nodes (white).

In should be noted that the boundary surfaces of the domain and body boundary surfaces are required when imposing boundary conditions in Equations (17) and (19), but no preparation of any kind of mesh is required. Each time-step fluid nodes that close to the walls is projected onto the geometry, as shown in Figure 2. The newly projected nodes impose Neumann (pressure gradient) boundary conditions in the PPE or Dirichlet (no-slip) boundary condition in the momentum equation. The projections guarantee that the closest fluid nodes are aligned along their (geometry) normals. This property of the LDD method allows for the accurate imposition of the Neumann boundary conditions.

## 4. Results and Discussion

### 4.1. Square Lid-Driven Cavity Flow

Lid-driven cavity tests are popular benchmarks for the newly introduced CFD solvers and methods [42]. This test’s simple geometry yields complex and distinct types of flows; therefore, it can provide a better understanding of the industrial complex processes in closed recirculating regions. While a strong extension occurs near the lid edges, the rotational flow can be seen in the center of the recirculating region. The ideally moving lid meets the stationary wall due to idealization, which results in a discontinuous velocity. It is hard to obtain the full range of kinematics, as well as rapid changes in pressure and stress near the corners, than it first appears. It is important to have a suitable method of calculating convection-dominated momentum transfer. For the above reasons, lid-driven flow in a cavity is a good initial experiment to validate flows for various Reynolds numbers and flow properties.

Casson fluid flow was simulated by the two-dimensional, steady-lid-driven cavity problem. The LDD numerical simulations were validated against the simulations conducted by [11]. The cavity space in dimensionless units is 1×1, and the moving lid had a steady, dimensionless velocity of $u_{lid}=1$. The no-slip condition was applied to the lid and wall boundaries. Two point cloud resolutions were tested. The time-step used for both resolutions was $\delta t=10^{-3}$, and the average time of the time-step calculation was 12ms on a modern GPU. 10s of actual physical time was simulated. The Reynolds number considered was $Re_{CA}=100$ and the Bingham number Bn=0.01, with the Reynolds and Bingham numbers, respectively, defined as follows:

(21) $$Re=\frac{\rho U_{\infty }l}{\mu_{\infty }},$$

(22) $$Bn=\frac{\tau_{y}l}{\mu_{\infty }U_{\infty }},$$

where ρ is the fluid density, $U_{\infty }$ is the velocity at infinite shear rate, *l* is the reference length, $\mu_{\infty }$ is the dynamic viscosity at infinite shear rate.

**Figure 3:**
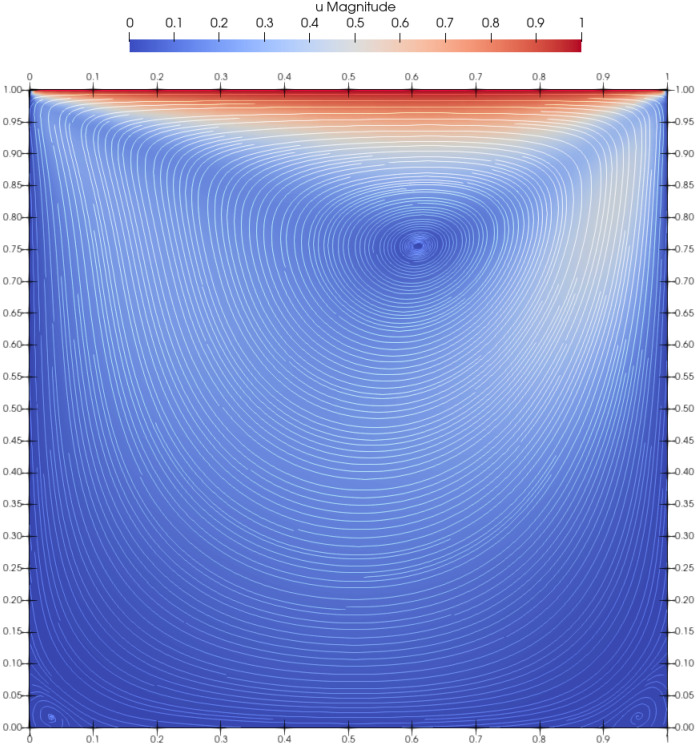
Plotted streamlines for Casson fluid flow at Re=100 and Bn=0.01.

**Figure 4:**
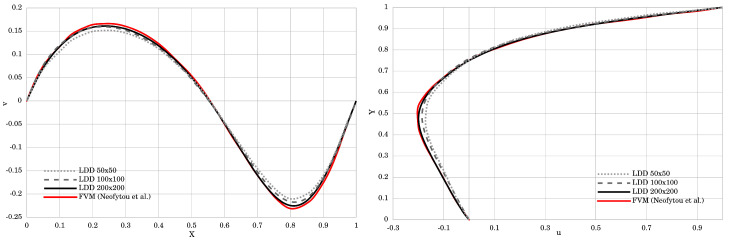
The velocity profile along the horizontal and vertical centerline of the square lid-driven cavity test.

Figure 3 renders the velocity magnitude and streamlines. The obtained results agree very well with those presented by Neofytou [11]. The plotted streamline pattern in Figure 3 showed that the center of the primary vortex was successfully and accurately captured, while the secondary vortices were also clearly reproduced in the bottom corners of the cavity box. Figure 4 plots the velocity profiles perpendicular to the vertical and horizontal centerlines of the cavity. Two simulations with different initial resolutions, 100×100 and 200×200, were performed to prove the convergence of the method. The *u*-component of the velocity for the finer resolution is in excellent agreement with the FEM method. The coarser resolution had slightly underestimated values, but overall agreed well with the referent results. The *v*-velocity plotted in the horizontal centerline of the cavity for both resolutions is in very good agreement with FEM. The only discrepancy occured at its highest and lowest peak, where the *v*-component of the velocity showed slightly lower absolute values, even though it agrees very well in all other areas.

### 4.2. Skewed Lid-Driven Cavity Flow

The Power Law viscosity model was tested for a fluid circulating in a lid-driven skewed cavity flow. Demirdžić et al. [43] was the first to publish results for skewed angles, $\alpha =30^{\circ }$ and $45^{\circ }$ for Newtonian fluids. Thohura et al. [39] compared their results with the published results and extended the investigation to various skew angles. An experiment of the skewed cavity using the Power Law, reported by Thohura et al. [39], is reproduced in this paper using the LDD method, and the results are compared.

The circulation pattern and vortex formation are highly dependent on the Reynolds number for any rheological behavior. Therefore, a relatively high Reynolds number was chosen as the simulation, Re=500, to show the stability and robustness of the LDD method. The Power Law index was n=1.5. The cavity in dimensionless units had a size of 1×1, and the angle of the side wall to the baseline was $\alpha =60^{\circ }$. The lid moved with a steady, dimensionless velocity of $u_{lid}=1$. The no-slip condition was applied to the lid and wall boundaries. Three initial resolutions of 50×50, 100×100 and 200×200 nodes were tested. The time step used for simulations was $\delta t=10^{-3}$, and the calculation of a step took 26ms in average. 20s of actual physical time was simulated until steady simulation was reached.

**Figure 5:**
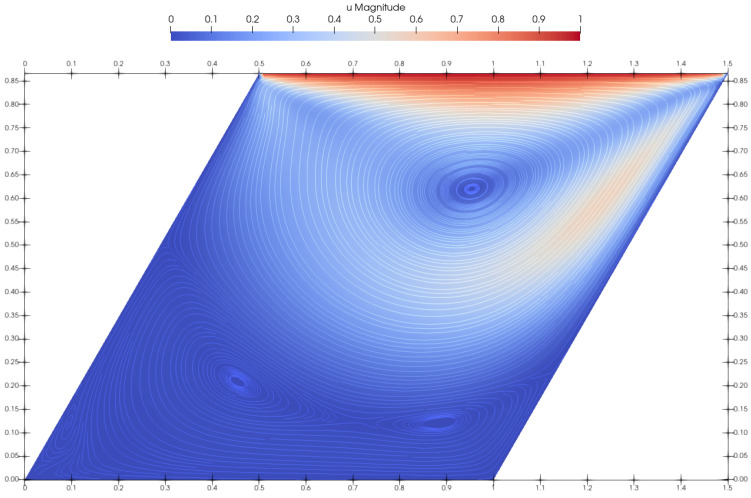
Velocity magnitude and streamlines for Power Law fluid flow in the skewed cavity, for Re=500 and n=1.5.

**Figure 6:**
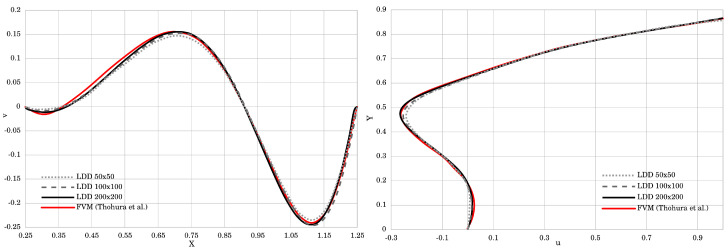
The velocity profile along the horizontal and vertical centerline of the skewed lid-driven cavity test.

In Figure 5, plotted streamlines correspond very well to the reference data provided by Thohura et al. [39]. The positions of the vortices are correctly captured, and vortex in the lower-right corner is more clearly represented in the LDD method than in the FVM. Figure 6 compares the simulated results of the *u* and *v* components of the velocity along the vertical and horizontal centerlines of the cavity with the referent data. For the *u*-component of the velocity, the plotted curves match almost perfectly, while *v*-component of the velocity has small discrepancy up to the height of Y=0.65. Overall, a good match between the obtained results is achieved, which proves that the method is capable of simulating non-Newtonian Power Law fluids.

### 4.3. Dam Break of a Bingham Fluid

**Figure 7:**
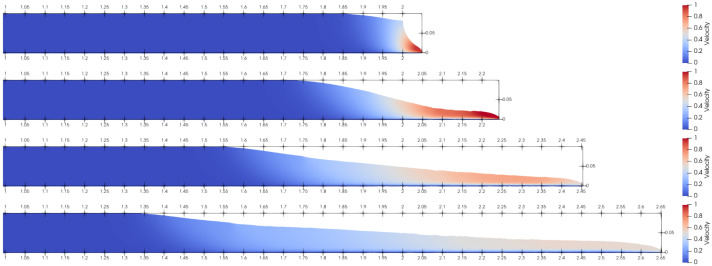
Dam break snapshots for time instances t= 0.1, 0.3, 0.6, 1.0 s, with the plotted velocity magnitude.

A dam break test was performed in accordance with the experimental parameters specified by Komatina and Jovanovíc [44]. A non-Newtonian fluid was represented by a mixture of water and clay in a reservoir released onto a channel with an initial length of 2 m and initial height of 0.1 m. The mixture density of water and mud was $\rho =1200\;\mathrm{kg}/m^{3}$, the Bingham yield stress was $\tau_{B}=25.0\;\mathrm{Pa}$ and the viscosity was $\mu_{B}=25.0\;\mathrm{Ns}/m^{2}$. The no-slip boundary condition was used along the channel walls. The simulation snapshots were taken at times t=0.1,0.3,0.6,1.0s, and rendered in Figure 7.

**Figure 8:**
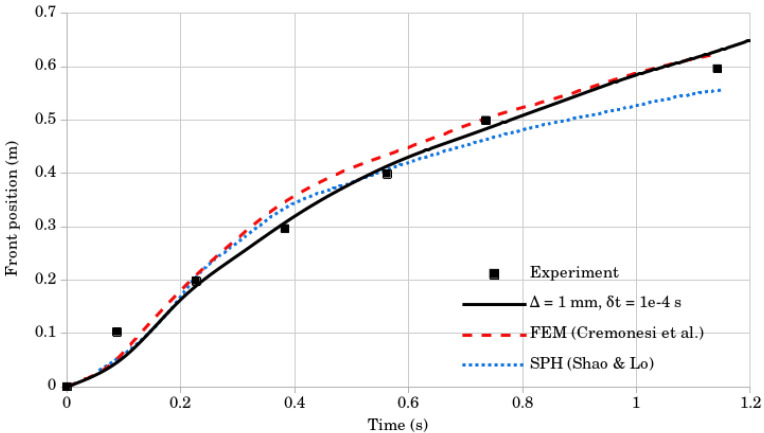
Mass front position results; numerical simulations compared to the experimental data.

The results of the LDD method were compared to the experimental data and other numerical methods in Figure 8. Shao and Lo [30] conducted the dam-break test by employing the Incompressible SPH (ISPH) method, while Cremonesi et al. [16] tested a Lagrangian formulation for weakly compressible fluids by employing an explicit solver based on PFEM. The simulated flow visually and numerically corresponded to the experimental data. The distinctive characteristics of the flow could be recognized. At the start of the flow, the free-standing end of the fluid column flowed, and the upper free corner began to collapse. Subsequently, the fluid tended to decrease in height as it flowed, obtaining peak velocity at the flow front. Towards the end, the flow behaved as a creeping flow and the surface profile was almost unchanged, which corresponds with the experimental findings. From the time instance t=0.0÷0.2s all the numerical methods showed a discrepancy that can be explained by the lack of a vertical wall in the channel that releases the flow. The numerical results of the LDD method are closer to the experimental results between t=0.2÷0.8s times than the other numerical methods. From t=0.8÷1.2s, the FEM method [16] and the LDD method both show a good fit with the experimental data. The results obtained by the LDD method clearly show the ability of the novel method to simulate Bingham-type flows and reproduce realistic results.

**Figure 9:**
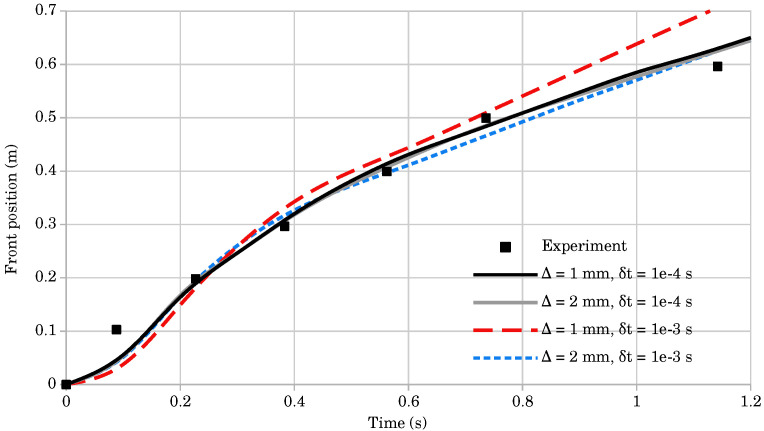
The evolution of mass front position for simulations with two node counts and time step sizes for the dam break test, compared to the experimental data.

A convergence study was performed using two simulations with different resolutions and time-step sizes. The first simulation used 200,000 nodes (Δ=1mm) and the second simulation used 50,000 nodes (Δ=2mm). For both resolutions, the tested time step sizes were $\delta t=10^{-3}$ and $\delta t=10^{-4}$. Table 1 shows the comparison of the tracked mass front position, obtained numerically and experimentally. The results are plotted in Figure 9. For the domain of 50,000 nodes, the average calculation time for one time-step $\delta t=10^{-3}$ took 35 ms, while for the domain of 200,000 nodes and time-step $\delta t=10^{-4}$, it took 200 ms. The results of the convergence study show that the domain of 200,000 nodes and the time-step $\delta t=10^{-4}$ gave the best results, and this was, therefore, used for the dam break comparison.

### 4.4. Fresh Concrete Slump Test

As a standard laboratory experiment, the slump test was used to determine the workability of fresh concrete. The slump test will be reproduced for the purpose of validating the method in three dimensions. Franci and Zhang [18] provided a detailed comparison of the experimental data and their numerical simulations.

The conical container was filled with concrete and the evolution of the form was measured after the container was removed. The spread and the slump of the evolving concrete were measured, i.e., fluid height and diameter. The test was completed once there was no movement in the fluid. This so-called Abram’s test [45] was computed using a single-phase Bingham model, with the regularization parameter m=1. Geometrical and material data are given in Table 2. The computation was made using 50,000 nodes in the domain and an average time-step took 55ms on the GPU. Time-step value was set to $\delta t=10^{-3}$ s, and 40s of physical time was simulated. To test the stability of the solver, the experiment was tested using larger time-steps, up to $\delta t=10^{-2}$ s, which remained stable.

**Figure 10:**
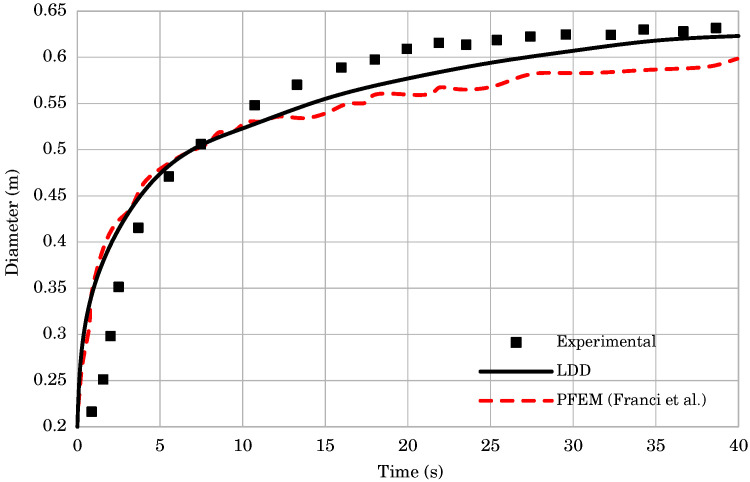
The evolution of the diameter for the Abram slump test. The results of the numerical simulations are compared to the experimental data.

**Figure 11:**
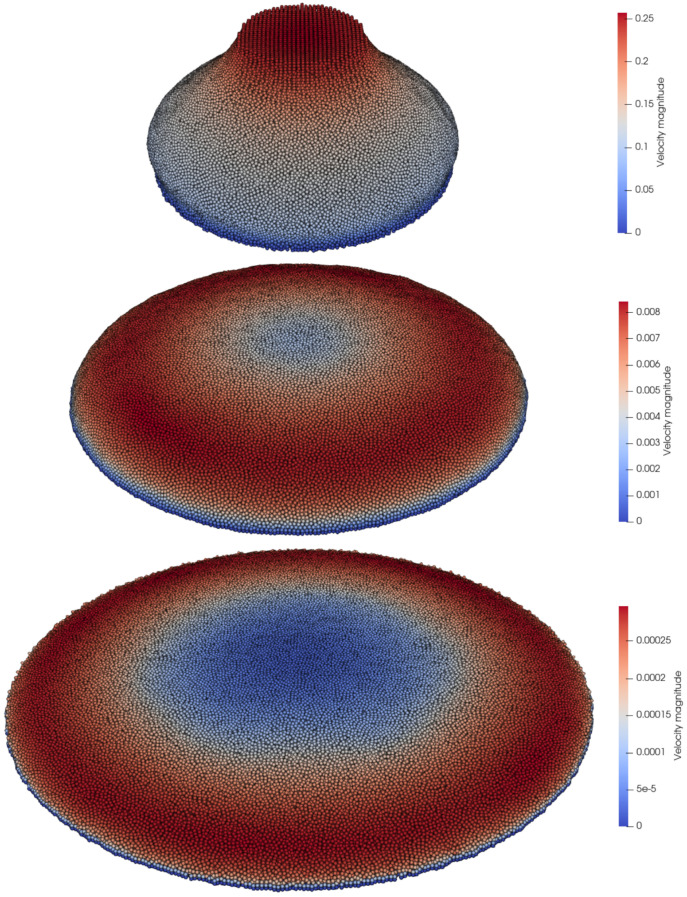
Simulation snapshots for three time instances t=0.5,5.0,40.0s with plotted velocity magnitudes.

The spread of cement was computed with good accuracy, as shown in Figure 10. During the time interval t=0.0÷10.0s, both PFEM and LDD numerical simulations resulted in approximately the same evolution of the diameter. The diameter values for those time instances are higher than in the experiment. After t=10.0s the results of the LDD and PFEM began to differ. They both showed a smaller diameter than the experiment, but the LDD method asymptotically reached the experimental results. At the time instance t=40.0s, the LDD method reached the experimentally obtained diameter, while the PFEM method still showed a lower value. The simulation snapshots presented in Figure 11 show the flow results for three time instances, t=0.5,5.0,40.0s. The axisymmetric flow reached maximum velocity at the top of the free surface, immediately after the container was removed. As the top slowly drops down, and the diameter evolves, the velocity decreases. After the cone completely collapses, the diameter slowly increases, until the final timepoint, where fluid has almost zero movement. These results are in agreement with those presented by Franci and Zhang [18].

## 5. Conclusions

In this paper, a novel meshless and Lagrangian method for 2D and 3D non-Newtonian problems with free-surface is introduced, named Lagrangian Differencing Dynamics (LDD). The method uses Lagrangian differences derived from finite differences to discretize and solve generalized Navier–Stokes equations. The fluid domain is represented by a cloud of points, in which each point advects in Lagrangian manner, while points near walls are projected to impose boundary conditions. Therefore, it is possible to simulate complex flows and complex shapes using relatively large time-steps. Due to the second-order consistent operators, and implicit solving of the pressure and velocity fields, the time-steps used were relatively large. In addition, parallelization on the CPU and GPU, as well the Lagrangian movement, allowed for simulations to be carried out relatively quickly.

Four tests with non-Newtonian fluids were validated using the Bingham, Casson and Power Law models. The results of the simulations are in good agreement with data from the corresponding experiments. The accuracy, high speed and robustness of the method are shown through different tests, parameters and flow types. For all of the performed benchmarks, the convergent behavior of the solver was verified. The velocity and pressure solvers showed stable and convergent behavior. It was noted that the regularization parameter *m* has an impact on the results, which will be investigated in future work.

Square and skewed cavity simulations were conducted in accordance with experiments and numerical simulations from the literature. The square lid-driven cavity test was simulated using the Casson model for Re=100, while the skewed lid-driven cavity was simulated using the Power Law model for Re=500. The problem of discontinuous velocity where the moving lid meets the stationary wall was determined, as well as the recirculation in the cavity. The full spectrum of kinematics and pressure fields was achieved. The distinctive characteristics of the flow were recognized in the dam break test simulated with the Bingham model. The Abram slump test was successfully reproduced and the evolution of the form agreed well with the experimental data.

Instead of the presented semi-implicit scheme, a completely implicit scheme for the velocity will be introduced in future work. Since the method is capable of reproducing accurate pressure fields, it will be validated by non-Newtonian models that significantly depend on the pressure. Future work will also address the validation of problems of Fluid–Structure Interaction (FSI). The current implementation of the introduced methodology is based on uniformly spaced nodes in the point-cloud, but the introduced operators handle non-uniform arrangements. Therefore, in future work, a local refining of the point cloud will be introduced to obtain more accurate solutions within turbulent areas of flow.

## Figures and Tables

**Table 1:** Mass front position obtained numerically and experimentally for six time instances.

Time (s)	Exp. Data	Mass Front (m), Δ=1 mm		Mass Front (m), Δ=2 mm	
		$\delta t=10^{-3}$ s	$\delta t=10^{-4}$ s	$\delta t=10^{-3}$ s	$\delta t=10^{-4}$ s
0.09	0.103	0.030	0.048	0.043	0.044
0.23	0.198	0.185	0.190	0.195	0.191
0.38	0.297	0.328	0.304	0.314	0.308
0.56	0.399	0.427	0.414	0.396	0.407
0.74	0.499	0.508	0.484	0.467	0.486
1.14	0.596	0.705	0.628	0.621	0.625

**Table 2:** The Abram slump test data.

$H_{0}$, m	$D_{0}$, m	$d_{0}$, m	ρ, kg/m${}^{3}$	μ, Pa·s	$\tau_{0}$, Pa
0.3	0.2	0.1	2200	255	32
